# Austin Flint, M. D., LL. D.

**Published:** 1886-04

**Authors:** 


### Austin Flint, M. D., LL.D.

The news of Dr. Flint's death, which took place Saturday, March 13th, as the result of an attack of apoplexy, has already been announced in all parts of the country, and the blow falls with staggering effect upon a profession that of late has lost many of its renowned and honored members. Dr. Flint died in the harness, stricken, as he had always hoped might be the case when his time should come, with a swiftly fatal and almost painless disease. After an illness of less than a day's duration, a great physician—one of the greatest that our country has produced—was translated from the busy exercise of his art to the peace and quiet of those who have loved their fellow-men.

Professor Flint was born in Petersham, Massachusetts, October 20, 1812, being descended from Thomas Flint, who having come from Derbyshire, in England, settled in Concord in 1638. For generations, the family has been of honorable renown in medicine, having furnished such men as Dr. Edward Flint, the great-grandfather, Dr. Austin Flint, the grandfather, and Dr. Joseph Henshaw Flint, the father of the late Professor Austin Flint. Dr. Flint received his general education partly at Amherst and partly at Harvard, and took his medical degree at the Harvard Medical School in 1833. Having practised for a time in Boston and in Northampton, he settled in Buffalo, N. Y., in 1836. During the first period of his residence in Buffalo, extending to the year 1844, he achieved a national reputation, and in the year mentioned he was appointed Professor of the Institutes and Practice of Medicine in Rush Medical College, of Chicago. He retained this position only a year, and then returned to Buffalo. There, in 1846, he founded the BUFFALO MEDICAL AND SURGICAI JOURNAL, a publication that still lives to do honor to his name; and the next year, in conjunction with Professor Frank H. Hamilton and the late Professor White, he took part in the founding of the Buffalo Medical College, in which institution he served as the Professor of Medicine until 1852, when he accepted the corresponding chair in the University of Louisville. In 1856 he again returned to Buffalo, and resumed his connection with the Buffalo Medical College as Professor of Pathology and Clinical Medicine. He spent the winters of 1858–'59, 1859–'60, and 1860–'61 in New Orleans, where he served as Physician to Charity Hospital and as Professor of Clinical Medicine. In 1859 he settled in New York, where, in 1861, he was appointed one of the physicians to Bellevue Hospital. He was one of the original members of the Faculty of the Bellevue Hospital Medical College, which held its first session in the season of 1861–'62, and in that institution he filled the chair of the Principles and Practice of Medicine and Clinical Medicine up to the time of his death. He had previously been made Professor of Pathology and Practical Medicine in the Long Island College Hospital, in Brooklyn, which position he resigned in 1868.

Dr. Flint's funeral, which took place at Christ Church, on Tuesday, March 16th, was attended by a great concourse of medical men, in their individual capacity or as the representatives of the larger medical societies. The number included many gentlemen from other cities. The remains were taken to Boston for interment.

During the days that they lay awaiting the obsequies, occurred the annual commencement of Bellevue Hospital Medical College. This occasion, ordinarily so joyful for all concerned, drew a tinge of gloom from the death of one of the great teachers of the school, but this was both tempered and hallowed by the tribute paid to Dr. Flint's memory by one of the speakers, Professor Dalton, the President of the College of Physicians and Surgeons

We can not at once realize the full influence of Dr. Flint's death upon professional matters. Above all its other aspects, of course, rises the fact that a great physician, a great teacher, and an earnest, noble-minded man has been lost to us. It is as an individual, and particularly as a consultant, on whom the local profession largely leaned, that he will most be missed; but the great school of which he was one of the founders, and to the success of which he contributed so conspicuously—and, indeed, medical teaching in general in this country, the quality of which has been distinctly colored both by his oral lectures and by his classical text-books—must necessarily require time to recover from the blow, but they will perpetually be the better for his having lived, and for many years to come they will bear manifest marks of his work. In another matter of great moment, Dr. Flint's death cannot fail to exert an important influence; he was to have presided over the Ninth International Medical Congress, having been chosen President both by the original committee of organization and by the enlarged committee which was charged, a year ago, with a revision of the work. Being, therefore, the choice of both parties to the unfortunate dispute that has arisen over the affairs of the Congress, he was the impersonation of the conservative force to which many in the profession, even among those who took so disheartened a view of the situation as to think it better that the proposed meeting of the Congress should not be held, looked as promising to prove the corrective of much that had gone wrong. A man of Dr. Flint's years would have been amply justified in declining to have anything further to do with an office which at the best is certain to be trying and wearing, and it is one of the foremost of his many titles to our gratitude that he remained patiently at his post, ready to take advantage of any tnrn of affairs that might enable him to compose the differences prevailing and pave the way for a successful meeting of the Congress.

No man ever reached a more honorable position in American medicine than was occupied by Dr. Flint for many years, which, perhaps, could not be more forcibly put than was done by the *Lancet* a few years ago, when, arguing against the narrow restrictions put upon medical practice in the United Kingdom, it characterized them as virtually shutting out such men as Trousseau and Flint. This position he achieved, too, without having made any noteworthy discoveries; it was rather his clear and precise way of dealing with the facts already at command —after all, one of the rarest of faculties—than any tinge given by him to the doctrine or practice of his time that won him his pre-eminence. This power of his was never more strikingly exemplified than when, toward the close of his life, he dealt with the bacillus theory of the ætiology of cholera in a paper which we published in our issue for October 25, 1884. Yet it cannot be said that he was not an original contributor to our knowledge; for it is difficult to imagine that anything like our present appreciation of cardiac murmurs, of differences of pitch in resonance, or of a multitude of other facts connected with the diagnosis of thoracic affections, could, but for him, have been reached for many years to come. Both in individual research and in the interpretation of others' work, then, he advanced our resources most abundantly, and he was assiduous in his kindness to his professional brethren and in upholding all measures that he conceived to be needful for the welfare of the body medical. The esteem in which he was held partook largely of the character of veneration, with no little admixture of tenderness, and every member of the American profession will feel his death as a personal loss.—*New York Medical Journal.*

